# The African Immigrant Dementia Education Project: A Community-University Partnership

**DOI:** 10.1093/geroni/igab046.1869

**Published:** 2021-12-17

**Authors:** Manka Nkimbeng, Wynfred Russell, Joseph Gaugler

**Affiliations:** 1 University of Minnesota, Minneapolis, Minnesota, United States; 2 African Career, Education & Resource Inc, Brooklyn Park, Minnesota, United States

## Abstract

The African Immigrant Dementia Education project is a community-university partnership with the goal of developing a culturally tailored dementia education program with African immigrants in Minnesota. In collaboration with our community partner (African Career, Education & Resource, Inc.), a project advisory board that features professionals and family members from the African immigrant community was assembled and its first meeting was held in February 2021. Preliminary discussions about content, mode of delivery and cultural considerations of an eventual dementia education intervention have begun. This presentation will offer details on the process of working with an advisory board and community partner to identify and culturally tailor an evidenced-based dementia education curriculum for a unique cultural group. Also, we will present challenges encountered during this process and offer suggestions and strategies to promote successful researcher-community partnerships.

